# Determinants of Progression to AIDS and Death Following HIV Diagnosis: A Retrospective Cohort Study in Wuhan, China

**DOI:** 10.1371/journal.pone.0083078

**Published:** 2013-12-23

**Authors:** Hongbo Jiang, Nianhua Xie, Beibei Cao, Li Tan, Yunzhou Fan, Fan Zhang, Zhongzhao Yao, Li Liu, Shaofa Nie

**Affiliations:** 1 Department of Epidemiology and Biostatistics, and the Ministry of Education Key Lab of Environment and Health, School of Public Health, Tongji Medical College, Huazhong University of Science and Technology, Wuhan, China; 2 Wuhan Center for Disease Control and Prevention, Wuhan, China; University of Texas Health Science Center San Antonio Texas, United States of America

## Abstract

**Objective:**

To identify determinants associated with disease progression and death following human immunodeficiency virus (HIV) diagnosis.

**Methods:**

Disease progression data from the diagnosis of HIV infection or acquiring immunodeficiency syndrome (AIDS) to February 29, 2012 were retrospectively collected from the national surveillance system databases and the national treatment database in Wuhan, China. Kaplan-Meier method, Logistic regression and Cox proportional hazards model were applied to identify the related factors of progression to AIDS or death following HIV diagnosis.

**Results:**

By the end of February 2012, 181 of 691 HIV infectors developed to AIDS, and 129 of 470 AIDS patients died among whom 289 cases received concurrent HIV/AIDS diagnosis. Compared with men infected through homosexual behavior, injection drug users possessed sharply decreased hazard ratio (*HR*) for progression to AIDS following HIV diagnosis [*HR* = 0.31, *95% confidence interval (CI),* 0.18–0.54, *P = *4.01×10^−5^]. HIV infectors at least 60 years presented 1.15-fold (*HR* = 2.15, *95% CI*, 1.15–4.03, *P* = 0.017) increased risk to develop AIDS when compared with those aged 17–29 years. Similarly, AIDS patients with diagnosis ages between 50 and 59 years were at a 1.60-fold higher risk of death (*HR* = 2.60, *95% CI*, 1.18–5.72, *P* = 0.017) compared to those aged 19–29 years. AIDS patients with more CD4^+^ T-cells within 6 months at diagnosis (cell/µL) presented lower risk of death (*HR* = 0.29 for 50- vs <50, *95% CI*, 0.15–0.59, *P* = 0.001). The highly active antiretroviral therapy (HAART) delayed progression to AIDS from HIV diagnosis (*HR* = 0.15, *95% CI*, 0.07–0.34, *P* = 6.46×10^−6^) and reduced the risk of death after AIDS diagnosis (*HR* = 0.02, *95% CI*, 0.01–0.04, *P* = 7.25×10^−25^).

**Conclusions:**

Progression to AIDS and death following HIV diagnosis differed in age at diagnosis, transmission categories, CD4^+^ T-cell counts and HAART. Effective interventions should target those at higher risk for morbidity or mortality, ensuring early diagnosis and timely treatment to slow down the disease progression.

## Introduction

Since China’s first acquired immunodeficiency syndrome (AIDS) case was identified in a dying tourist in 1985 [1], human immunodeficiency virus (HIV) had spread in the country and became a complex and challenging public health concern. At the end of 2011, about 780,000 patients were living with HIV (PLHIV) in China while the estimated number of new infections and death in 2011 was 48,000 and 28,000, respectively [2].

In order to provide HIV treatment and care primarily for rural and low-income urban patients who met the national treatment guidelines, the National Free Antiretroviral Treatment Program (NFATP) was initiated in October 2002 and subsequently expanded nationwide in 2003 [3]. At the end of 2011, a total of 3,142 antiretroviral therapy (ART) providers were available nationwide, located in 2,082 counties (or districts) among 31 provinces (and autonomous regions, municipalities). The total number of people ever receiving and currently receiving treatment increased from 81,739 and 65,481 in 2009 to 155,530 and 126,448 in 2011, respectively [2]. With the advent of highly active antiretroviral therapy (HAART), progression from HIV diagnosis to AIDS has been slowed down substantially, and so has the progression to death following AIDS diagnosis [4], [5], [6], [7], [8].

Despite this dramatic achievement, there remain questions about whether survival differs in specific subpopulations and what factors may drive such variation. Previous studies have suggested that age, gender, transmission category, educational level, marital status, and CD4^+^ T-cell count [4], [9], [10], [11], [12] might be markers for differences in access to treatment, adherence, or disease severity at diagnosis, ultimately leading to disparities in survival. Moreover, it would also be meaningful work to find out factors [13] which may be associated with progression to AIDS. However, most of these observations have been made from western countries and the corresponding situation in China is far from clear. Besides, little evidence has addressed the determinants associated with AIDS progression after HIV diagnosis [9], [14]. Against this background, we used data from Wuhan Center for Disease Control and Prevention (CDC) through the national surveillance system and the national treatment database to determine the related factors of progression to AIDS or death following HIV diagnosis in Wuhan, China, aiming to attain better understanding about the disease progression in developing countries.

## Methods

Subjects recruited in this study were identified through the two national databases, namely national surveillance system and national treatment database. In China, all HIV/AIDS cases diagnosed are compulsorily reported to CDC through the national surveillance system, which was upgraded to China Information System for Disease Control and Prevention (CISDCP) in 2003 [15]. The national treatment database which was established in late 2004, including data on current patients who met the national treatment criteria and those treated before 2004 [3], [16], [17], [18]. The national treatment criteria referred to World Health Organization disease stage III or IV, CD4^+^ T-cell count <200 cells/µL (increasing to <350/µL in 2008), or total lymphocyte count <1200 cells/µL. HIV infectors and AIDS patients were followed up with a face-to-face interview every six months and three months, respectively. After each visit, local health care workers completed a standardized case report form (CRF) and faxed the form to the Chinese CDC via Datafax (Clinical DataFax Systems) [18]. These CRFs were then maintained in an ongoing observational treatment database [3]. Wuhan, the capital city of Hubei province in central China, had built the web-based reporting system of conventional infectious disease situation network, and realized on-line direct report infectious disease situation in September 2003, and then upgraded the system to special reporting system of AIDS and tuberculosis in 2005 [19]. Before 2003, the information of HIV/AIDS cases was recorded and then sent to Wuhan CDC via emails. After the reporting system was built, the information was inputted into the system by the staff of Wuhan CDC. Data obtained from the epidemiological database of the national surveillance system included demographic characteristics, transmission category, date of diagnosis, date of death (if applicable), and data from the national treatment database included date of treatment, drug regimens and so on.

### Ethics Statement

The written consents were obtained from the Wuhan Center for Disease Control and Prevention which declared their willing to participate in the study and the data relating to disease progression of HIV/AIDS cases can be extracted from the national surveillance system databases and the national treatment database. All information was de-identified and only aggregated data were used for data analysis. The study was approved by the institutional review board of Tongji Medical College of Huazhong University of Science and Technology.

### HIV Diagnosis to AIDS

We used data to determine disease progression to AIDS following HIV diagnosis among adolescents and adults (≥13 years old at diagnosis). We included subjects who (1) were Wuhan residents and had been reported by Wuhan CDC, (2) were confirmed to be HIV-infected by a positive Western blot result, and (3) were aged older than 13 years at diagnosis. Given difference in disease progression between adolescents and adults, we excluded infectors who were infected perinatally or younger than 13 years of age at diagnosis [20]. Cases were followed up from the date of HIV diagnosis until February 29, 2012 (i.e., censoring date). It was assumed that the disease did not progress to AIDS for subjects who were free of AIDS diagnosis report at censoring date. Persons who died during the follow-up period were censored at date of death. Of 695 HIV infectors met the inclusion criteria above, 4 (0.58%) were excluded from the analyses because of too much missing information.

### AIDS Diagnosis to Death

We also determined survival among adolescents and adults (≥13 years old) diagnosed with AIDS. Subjects who met the inclusion criteria in the part of HIV diagnosis to AIDS, and received a diagnosis of AIDS either by an AIDS-defining illness or by having a CD4^+^ T-cell count <200 cells/µL before February 29, 2012 were included. Patients were followed up from the date of AIDS diagnosis until February 29, 2012. Individuals free of death at censoring date were assumed to be alive. Patients who died not due to AIDS or AIDS-defining illnesses during the follow-up period were censored at date of death. Of 472 AIDS cases met the criteria above, 2 (0.42%) were excluded from the analyses because of too much missing information.

### Data Analysis

The primary end point of the AIDS-related mortality was death attributable to AIDS or AIDS-defining illnesses. By using the overall period 1999–2002 as a whole owing to the small numbers of annual AIDS-related death during these years, then yearly from 2003 to 2011, the AIDS-related mortality was defined as the total number of subjects who died of AIDS or AIDS-defining illnesses within the specified time period divided by the sum of person-years for individuals who received a diagnosis of AIDS during the same period. The year that a person was lost to follow-up or died of other causes (e.g., other disease, suicide, accident et al) was counted as one-half of a person-year. A similar method was conducted to calculate each individual’s person-years receiving HAART during each time period. The Log-linear model was used to determine whether a decreasing trend for mortality existed.

Kaplan-Meier method was used in STATA software (Stata Corp., College Station, TX) to calculate the proportions of HIV infectors who progressed to AIDS and relative survival proportions for AIDS patients. Stratified analyses were conducted by sex, age at AIDS diagnosis or HIV diagnosis, transmission category, marital status, educational level, receiving HAART or not, CD4^+^ T-cell count at AIDS diagnosis (first record within 6 months at diagnosis), and concurrent diagnosis of HIV diagnosis and AIDS or not (only for AIDS cases). The cross-table was applied to assess the linear trend in morbidity and mortality among strata of selected variables.

We used non-conditional Logistic regression analysis and Cox proportional hazard model to address potential risk factors of AIDS morbidity and mortality [21]. Univariate and multivariate non-conditional Logistic regression analyses were conducted to explore the potential factors associated with the likelihood of AIDS events or death events. Univariate Cox models were applied to assess the unadjusted relationship between morbidity or mortality and specified covariates of interest. Multivariate Cox regression analysis was conducted after adjustment for sex and factors statistically significant in univariate analysis. Data were analyzed using SPSS, version 12.0 (SPSS. Inc., USA). All hypothesis testing was based on 2-sided tests with *α* = 0.05.

## Results

### HIV Diagnosis to AIDS

As reported to the Wuhan CDC, 691 persons received HIV diagnosis from 1994 to February 2012, of whom 181 HIV infectors developed to AIDS. 11 (1.59%) HIV infectors were lost to follow up. 456 (65.99%) HIV infectors did not progress to AIDS and were still alive, while 43 (6.22%) cases died before they progressed to AIDS. The majority of HIV infectors were male (85.14%) and attained secondary education (60.20%). 679 (98.26%) HIV infectors were Han Chinese, and the other 12 were minorities. The median age at HIV diagnosis was 34 years old, ranging from 17 to 85 years old [interquartile range (IQR): 26–46 years old]. Men who had sex with men (MSMs) accounted for 48.63%, followed by heterosexual contact (HC) with someone at high risk or with a diagnosis of HIV infection or AIDS, injection drug users (IDUs), infection through Blood (blood transfusion or blood products), and other unknown reasons (Table 1). The single persons accounted for 48.48%, and only 17.66% of HIV infectors received HAART.

**Table 1:** Percentage of HIV infectors and who progressed to AIDS, Wuhan, China.

Characteristics	Number Entering Interval	%	Percent with AIDS 1 year		Number Entering Interval	Percent with AIDS 3 year	
			after HIV diagnosis			after HIV diagnosis	
			%	*95% CI*		%	*95% CI*
**Sex**							
female	102	14.76	22.93	15.41–33.33	36	45.59	34.04–58.96
male	589	85.24	17.01	13.76–20.94	153	35.19	29.69–41.36
**Age(years)**							
17–*	262	37.92	14.30	9.88–20.46	67	32.12	23.65–42.66
30–	174	25.18	19.66	14.13–27.00	59	34.10	26.36–43.35
40–	131	18.96	22.63	15.71–31.96	48	37.19	27.35–49.17
50–	76	11.00	10.57	4.79–22.44	10	73.86	49.05–93.07
≥60	48	6.95	33.98	18.85–56.21	5	69.19	43.67–91.07
**Transmission category**							
MSM	336	48.63	17.86	13.17–23.98	50	41.76	31.34–54.03
HC	243	35.17	21.45	16.34–27.89	73	43.47	35.22–52.73
Blood	26	3.76	27.56	13.42–51.38	7	73.25	49.73–92.01
IDU	77	11.14	9.48	4.63–18.88	55	16.83	9.91–27.78
Unknown/other	9	1.30			4		
**Marital status**							
Single	335	48.48	16.37	12.15–21.88	85	28.42	21.8–36.54
Married	174	25.18	25.07	18.51–33.43	42	52.04	41.3–63.7
Widowed/divorced	182	26.34	14.92	10.14–21.69	62	37.21	28.61–47.41
**Education** **							
≤Primary	63	9.12	23.32	13.52–38.44	20	37.87	24.61–55.14
Secondary	416	60.20	18.19	14.17–22.75	120	40.74	34.35–47.83
≥College/university	204	29.52	15.60	10.18–23.51	44	26.90	17.83–39.35
Unknown/other	8	1.16			5		
**HAART**							
No	569	82.34	21.00	17.41–25.21	164	41.92	36.39–47.94
Yes	122	17.66	4.30	1.59–11.33	25	6.25	2.52–15.07
**Total**	691	100.00	17.90	14.84–21.49	189	37.24	32.15–42.87

Data reported to Centers for Disease Control and Prevention from 1994 to February 29, 2012.

* The youngest person who met the inclusion criteria was 17 years old.

** ≤Primary: illiterate or primary school, Secondary: middle school, high school or technical secondary school.

MSM = men who had sex with men; IDU = injection drug user; HC = heterosexual contact with a high-risk individual or person with HIV infection or AIDS; Blood = blood transfusion or blood products; AIDS = acquired immunodeficiency syndrome; HIV = human immunodeficiency virus; CI = confidence interval.

The mean follow-up time of HIV infectors was 18.58±20.16 months. 17.90%, 28.15% and 57.27% of HIV infectors progressed to AIDS after one year, two years, and five years, respectively. The rate of progression to AIDS within one or three years following HIV diagnosis was similar between males and females, and it was lower for individuals receiving HAART than those did not. The proportion of HIV infectors with progression to AIDS presented no difference in age at diagnosis, transmission categories, marital statuses, educational level within one year following diagnosis. Otherwise, different proportion of HIV infectors with progression to AIDS arose among the groups mentioned above except educational level at three years (Table 1).

Results of univariate and multivariate non-conditional Logistic regression analyses are presented in Table 2. In the univariate analyses, sex, age at diagnosis, transmission categories, marital status, educational level and HAART were statistically significant associated with AIDS events (*P*<0.05 for all). After mutual adjustment by these factors, sex, age at diagnosis, and marital status were no longer statistically significant associated with AIDS events (*P*>0.05 for all). Otherwise, HIV infectors transmitted by HCs and Blood were more likely to develop AIDS compared to MSMs [*Odds ratio* (*OR*) = 1.81 for HCs vs MSMs, *95% CI*, 1.13–2.89, *P* = 0.013; *OR* = 4.76 for Blood vs MSMs, *95% CI*, 1.89–12.01, *P* = 0.001]. The more educated HIV infectors and those under HAART were less likely to develop AIDS (*OR* = 0.52 for ≥College/university vs Secondary, *95% CI*, 0.32–0.85, *P* = 0.010; *OR* = 0.13 for HAART vs no HAART, *95% CI*, 0.05–0.30, *P* = 2.61×10^−6^).

**Table 2:** ORs and 95% CIs of likelihood to develop AIDS among HIV infected persons, non-conditional Logistic regression analysis model results according to demographic factors and selected variables at HIV diagnosis, Wuhan, China.

Characteristics	Persons with HIV diagnosis			
	*OR* (*95% CI*)	*P* value	Adjust *OR* (*95% CI*)*	*P* value
**Sex**		0.016		0.472
male	1.00		1.00	
female	0.46 (0.30–0.72)		0.82 (0.49–1.30)	
**Age(years)**		0.016		0.275
17–**	1.00		1.00	
30–	2.02 (1.30–3.13)	0.002	1.59 (0.94–2.69)	0.085
40–	1.75 (1.08–2.84)	0.023	1.11 (0.94–2.07)	0.741
50–	1.28 (0.70–2.37)	0.423	0.93 (0.43–2.00)	0.853
≥60	2.07 (1.01–4.06)	0.034	0.87 (0.38–1.98)	0.737
**Transmission category**		3.82×10^−7^		0.002
MSM	1.00		1.00	
HC	2.63 (1.79–3.88)	1.01×10^−6^	1.81 (1.13–2.89)	0.013
Blood	6.67 (2.91–15.28)	7.10×10^−6^	4.76 (1.89–12.01)	0.001
IDU	1.96 (1.11–3.46)	0.021	0.95 (0.51–1.79)	0.883
Unknown/other	1.40 (0.28–6.91)	0.681	0.57 (0.10–3.27)	0.529
**Marital status**		1.68×10^−4^		0.538
Single	1.00		1.00	
Married	2.29 (1.51–3.46)	8.98×10^−5^	1.38 (0.78–2.45)	0.266
Widowed/divorced	1.88 (1.24–2.85)	0.003	1.24 (0.71–2.17)	0.459
**Education** ****		2.26×10^−5^		0.044
≤Primary	1.00		0.99 (0.53–1.84)	0.970
Secondary	0.80 (0.46–1.40)	0.435	1.00	
≥College/university	0.31 (0.16–0.59)	3.97×10^−4^	0.52 (0.32–0.85)	0.010
Unknown/other	3.11 (0.68–14.23)	0.144	2.48 (0.47–12.98)	0.283
**HAART**		5.19×10^−7^		2.61×10^−6^
No	1.00		1.00	
Yes	0.12 (0.05–0.27)		0.13 (0.05–0.30)	

Data reported to Centers for Disease Control and Prevention from 1994 to February 29, 2012.

* Adjusted for factors statistically significant in univariate analysis.

** The youngest person who met the inclusion criteria was 17 years old.

**** ≤Primary: illiterate or primary school, Secondary: middle school, high school or technical secondary school.

MSM = men who had sex with men; IDU = injection drug user; HC = heterosexual contact with a high-risk individual or person with HIV infection or AIDS; Blood = blood transfusion or blood products; AIDS = acquired immunodeficiency syndrome; HIV = human immunodeficiency virus; OR = odds ratio; CI = confidence interval.

Table 3 shows the results of the univariate and multivariate Cox regression analysis of factors associated with the risk of progressing to AIDS. The univariate analysis demonstrated that factors, including age at diagnosis, transmission categories, marital status, educational level and HAART, had significant influence on AIDS progression. Age might be a risk factor for developing AIDS (*P* for trend = 0.048), whereas education turned out to be a protective factor (*P* for trend <0.001). The results of the multivariate analysis after adjustment for sex and factors statistically significant in univariate analysis showed that IDUs were less likely to develop AIDS following HIV diagnosis (*HR* = 0.31; *95% CI*, 0.18–0.54, *P = *4.01×10^−5^) compared with MSMs. The individuals aged ≥60 years had a 1.15-fold higher risk of progressing to AIDS (*HR* = 2.15, *95% CI,* 1.15–4.03, *P* = 0.017) compared with those aged 17–29 years. The HAART reduced substantially the risk of progression to AIDS (*HR* = 0.15, *95% CI,* 0.07–0.34, *P = *6.46×10^−6^).

**Table 3:** HRs and 95% CIs of progressing to AIDS among HIV infectors, Cox proportional hazard model results according to demographic factors and selected variables at HIV diagnosis, Wuhan, China.

Characteristics	Persons with HIV diagnosis			
	*HR* (*95% CI*)	*P* value	Adjust *HR* (*95% CI*)*	*P* value
**Sex**		0.200		0.560
male	1.00		1.00	
female	0.79 (0.55–1.13)		0.89 (0.59–1.33)	
**Age(years)**		0.005		0.163
17–**	1.00		1.00	
30–	1.30 (0.89–1.90)	0.178	1.47 (0.96–2.26)	0.079
40–	1.33 (0.88–2.02)	0.180	1.35 (0.83–2.20)	0.228
50–	1.53 (0.89–2.63)	0.123	1.59 (0.85–2.96)	0.145
≥60	2.97 (1.69–5.21)	1.56×10^−4^	2.15 (1.15–4.03)	0.017
*χ^2^* for trend	3.93	0.048		
**Transmission category**		9.25×10^−5^		3.96×10^−5^
MSM	1.00		1.00	
HC	1.28 (0.91–1.81)	0.162	0.88 (0.59–1.30)	0.508
Blood	2.24 (1.26–3.99)	0.006	1.60 (0.86–2.96)	0.137
IDU	0.51 (0.30–0.85)	0.010	0.31 (0.18–0.54)	4.01×10^−5^
Unknown/other	0.63 (0.15–2.60)	0.525	0.39 (0.09–1.71)	0.213
**Marital status**		0.001		0.188
Single	1.00		1.00	
Married	1.91 (1.35–2.76)	2.91×10^−4^	1.35 (0.86–2.12)	0.192
Widowed/divorced	1.36 (0.95–1.95)	0.094	0.97 (0.62–1.51)	0.886
**Education** ***		0.124		0.379
≤Primary	1.00		1.00	
Secondary	0.85 (0.54–1.35)	0.495	0.97 (0.60–1.56)	0.901
≥College/university	0.56 (0.32–0.99)	0.044	0.66 (0.36–1.21)	0.181
Unknown/other	1.20 (0.45–3.18)	0.710	0.97 (0.35–2.64)	0.951
*χ^2^* for trend****	18.88	<0.001		
**HAART**		2.28×10^−5^		6.46×10^−6^
No	1.00		1.00	
Yes	0.17 (0.08–0.39)		0.15 (0.07–0.34)	

Data reported to Centers for Disease Control and Prevention from 1994 to February 29, 2012.

* Relative risk adjusted for sex and the other factors statistically significant in univariate analysis.

** The youngest person who met the inclusion criteria was 17 years old.

*** ≤Primary: illiterate or primary school, Secondary: middle school, high school or technical secondary school.

**** Unknown exclude.

MSM = men who had sex with men; IDU = injection drug user; HC = heterosexual contact with a high-risk individual or person with HIV infection or AIDS; Blood = blood transfusion or blood products; AIDS = acquired immunodeficiency syndrome; HIV = human immunodeficiency virus; HR = hazard ratio; CI = confidence interval.

### AIDS Diagnosis to Death

470 persons received AIDS diagnosis from 1999 to February 2012, of whom 129 died, including 27 censored patients who died not due to AIDS or AIDS defining diseases. 5 (1.06%) AIDS patients were lost to follow up, and 336 (71.49%) AIDS patients survived. 289 of 470 (61.46%) AIDS patients received concurrent HIV/AIDS diagnosis. The majority of AIDS patients were male (75.32%) and attained secondary education (66.38%). 454 (96. 06%) AIDS patients were Han Chinese and the other 16 were minorities. The median age at AIDS diagnosis was 39 years old, ranging from 19 to 79 years old (IQR: 31–49 years old). The married patients accounted for 42.98%, and over half (52.12%) of the patients with AIDS were infected through HC (Table 4). AIDS Patients with low CD4^+^ T-cell counts (<50 cells/µL) accounted for 23.4%, and 68.94% of patients received HAART.

**Table 4:** Relative survival of persons with a diagnosis of AIDS in Wuhan, China.

Characteristics	NumberEnteringInterval	%	Percent surviving 1 year		Number Entering Interval	Percent surviving 3 year	
			after AIDS diagnosis			after AIDS diagnosis	
			%	*95% CI*		%	*95% CI*
**Sex**							
female	116	24.68	83.49	74.76–89.41	49	74.36	63.39–82.49
male	354	75.32	82.91	77.96–86.85	111	79.90	74.14–84.51
**Age(years)**							
19–*	101	21.49	85.13	76.11–90.95	34	76.75	63.16–85.87
30–	146	31.06	91.37	84.88–95.16	65	87.42	79.26–92.52
40–	108	22.98	78.20	68.36–85.31	32	74.35	63.30–82.52
50–	79	16.81	70.49	57.01–80.45	24	66.78	51.82–78.04
≥60	36	7.66	88.86	69.27–96.27	5	79.97	51.04–92.84
**Transmission category**							
MSM	120	25.53	95.23	87.43–98.23	23	95.23	87.43–98.23
HC	245	52.13	80.85	74.75–85.62	86	75.34	67.95–81.26
Blood	57	12.13	71.60	57.37–81.80	30	67.37	52.79–78.33
IDU	39	8.30	86.50	67.66–94.76	18	77.13	55.26–89.25
Unknown/other	9	1.91			3		
**Marital status**							
Single	135	28.72	90.00	82.87–94.26	43	85.64	75.45–91.83
Married	202	42.98	78.00	70.84–83.60	74	73.05	64.94–79.58
Widowed/divorced	133	28.30	83.69	75.20–89.47	43	78.11	67.33–85.70
**Education** **							
≤Primary	66	14.04	74.52	61.23–83.83	28	67.82	53.51–78.58
Secondary	312	66.38	83.17	77.89–87.30	101	79.23	72.93–84.23
≥College/university	83	17.66	93.09	83.85–97.13	26	93.09	83.85–97.13
Unknown/other	9	1.91			5		
**CD4^+^ T–cell count (cell/µL)**							
<50	110	71.38	70.53	61.13–79.38	37	63.25	51.04–73.20
50–	178	91.77	91.94	84.46–95.42	56	89.01	81.24–93.68
≥200	60	100.00	97.75	–	21	100.00	–
Unknown	122	71.38			46		
**AIDS at HIV infection diagnose**							
No	181	91.31	89.51	85.32–94.93	61	87.72	79.18–92.91
Yes	289	77.75	70.97	71.89–82.54	99	72.03	65.21–77.73
**HAART**							
No	146	34.79	34.96	24.16–45.61	13	21.50	12.23–32.49
Yes	324	97.55	97.39	94.91–98.83	147	95.28	90.96–97.57
**Total**	470	100.00	83.15	79.02–86.54	160	78.16	73.06–82.41

Data reported to Centers for Disease Control and Prevention from 1999 to February 29, 2012.

* The youngest person who met the inclusion criteria was 19 years old.

** ≤Primary: illiterate or primary school, Secondary: middle school, high school or technical secondary school.

MSM = men who had sex with men; IDU = injection drug user; HC = heterosexual contact with a high-risk individual or person with HIV infection or AIDS Blood = blood transfusion or blood products; AIDS = acquired immunodeficiency syndrome; HIV = human immunodeficiency virus; CD4^+^ T-cell count = CD4^+^ T-cell count within 6 months at diagnosis (cell/µL); CI = confidence interval.

**Figure 1:**
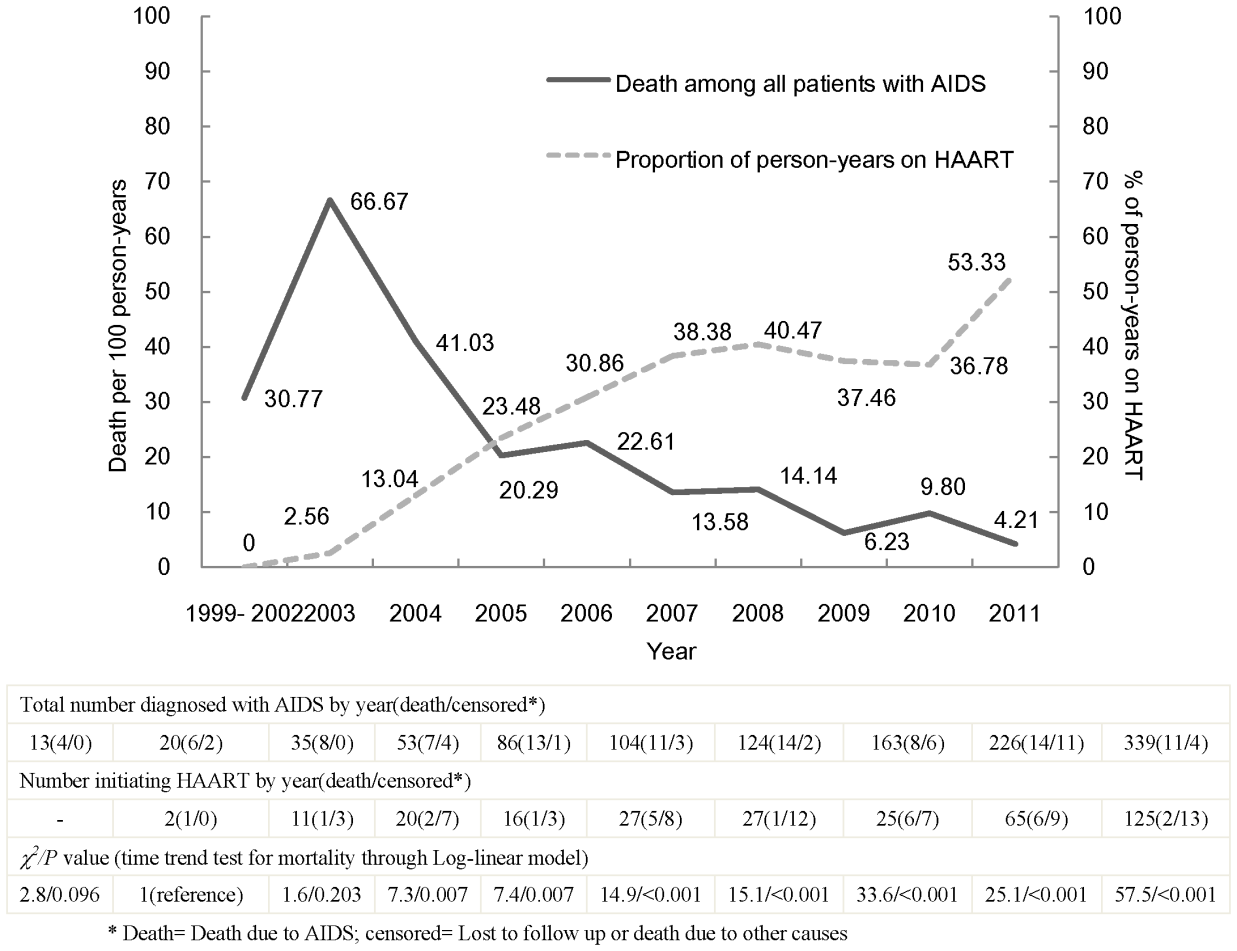
Mortality over time for AIDS patients included in the study.

The AIDS-related mortality over time among AIDS patients are shown in Figure 1. The NFATP began in 2003 in Wuhan, and thereafter increased its capacity. Compared with the AIDS-related mortality of 66.67/100 person-years in 2003, the data decreased statistically significantly each year from 2005 onward (*P*<0.05). Conversely, the percentage of AIDS patients receiving HAART increased from 2.56% of person-years in 2003 to 53.33% in December 2011, presenting an inverse relationship with AIDS-related mortality.

The mean follow-up time of AIDS patients was 23.47±26.93 months. 83.15%, 81.03% and 74.86% of AIDS patients survived after one year, two years and five years, respectively. A proportion of 71.46% presented for a 10-year-survival by the end of observation. Survival one or more years after AIDS diagnosis was similar between males and females. And the one or more years’ survival proportion of AIDS patients did not differ by marital statuses. However, differential survival after AIDS diagnosis arose among age at diagnosis, transmission categories, educational level, CD4^+^ T-cell counts within 6 months at diagnosis (cell/µL), concurrent HIV/AIDS diagnosis and receiving HAART (Table 4).

Results of univariate and multivariate non-conditional Logistic regression analyses are presented in Table 5. In the univariate analyses, sex, age at diagnosis, transmission categories, marital status, educational level, CD4^+^ T-cell count within 6 months at diagnosis, concurrent HIV/AIDS diagnosis, and HAART were statistically significant associated with death events (*P*<0.05 for all). After mutual adjustment by these factors, sex, age at diagnosis, transmission categories, marital status and concurrent HIV/AIDS diagnosis were no longer statistically significant associated with death events (*P*>0.05 for all). Otherwise, the more educated AIDS patients and those under HAART were less likely to die of AIDS or AIDS-defining illnesses (*OR* = 0.04 for Secondary vs ≤Primary, *95% CI*, 0.003–0.47, *P* = 0.011; *OR* = 0.02 for ≥College/university vs ≤Primary, *95% CI*, 0.002–0.26, *P* = 0.002; *OR* = 0.02 for HAART vs no HAART, *95% CI*, 0.01–0.05, *P = *1.90×10^−19^). Patients with more CD4^+^ T-cell count within 6 months at diagnosis was associated with less likelihood to die of AIDS or AIDS-defining illnesses (*OR* = 0.30 for 50- vs <50, *95% CI*, 0.11–0.80, *P* = 0.017; *OR* = 0.07 for ≥200 vs <50, *95% CI*, 0.01–0.68, *P* = 0.022).

**Table 5:** ORs and 95% CIs of likelihood to die of AIDS or AIDS-defining illnesses among AIDS patients, non-conditional Logistic regression analysis model results according to demographic factors and selected variables at AIDS diagnosis, Wuhan, China.

Characteristics	Persons with AIDS diagnosis*			
	*OR* (*95% CI*)	*P* value	Adjust *OR* (*95% CI*)**	*P* value
**Sex**		0.212		0.615
male	1.00		1.00	
female	0.73 (0.45–1.19)		1.26 (0.51–3.07)	
**Age(years**)		0.022		0.414
19–***	1.00		1.00	
30–	0.77 (0.39–1.50)	0.438	1.51(0.31–7.65)	0.596
40–	1.30 (0.67–2.54)	0.443	2.14(0.25–3.70)	0.964
50–	2.24 (1.13–4.43)	0.020	2.14 (0.57–8.06)	0.260
≥60	1.44 (0.58–3.55)	0.431	2.25 (0.58–8.82)	0.244
**Transmission category**		3.55×10^−5^		0.543
MSM	1.00		1.00	
HC	7.79 (3.04–19.96)	1.88×10^−5^	0.96 (0.10–8.84)	0.972
Blood	13.42 (4.72–38.14)	1.11×10^−6^	1.76 (0.26–11.97)	0.562
IDU	7.93 (2.52–25.00)	4.08×10^−4^	3.16 (0.38–26.24)	0.286
Unknown/other	18.40 (3.75–90.29)	3.33×10^−4^	1.05 (0.13–8.53)	0.966
**Marital status**		0.001		0.928
Single	1.00		1.00	
Married	3.07 (1.68–5.61)	2.72×10^−4^	1.27 (0.36–4.51)	0.708
Widowed/divorced	1.89 (0.97–3.71)	0.062	1.11 (0.48–2.59)	0.806
**Education** ****		2.93×10^−5^		0.010
≤Primary	1.00		1.00	
Secondary	0.52 (0.29–0.92)	0.026	0.04 (0.003–0.47)	0.011
≥College/university	0.12 (0.04–0.34)	6.01×10^−5^	0.02 (0.002–0.26)	0.002
Unknown/other	3.74 (0.86–16.35)	0.080	0.01 (0.001–0.15)	0.001
**CD4^+^ T–cell count (cell/µL)**		2.60×10^−11^		0.001
<50	1.00		1.00	
50–	0.21 (0.11–0.42)	6.33×10^−6^	0.30 (0.11–0.80)	0.017
≥200	0.04 (0.01–0.30)	0.002	0.07 (0.01–0.68)	0.022
Unknown	1.79 (1.04–3.08)	0.035	1.51 (0.63–3.66)	0.358
**AIDS at HIV infection diagnosis**		2.86×10^−6^		0.133
No	1.00		1.00	
Yes	3.17 (2.14–6.43)		1.91 (0.82–4.46)	
**HAART**		5.29×10^−28^		1.90×10^−19^
No	1.00		1.00	
Yes	0.02 (0.01–0.04)		0.02 (0.01–0.05)	

Data reported to Centers for Disease Control and Prevention from 1999 to February 29, 2012.

* HIV infection diagnosed with or without a concurrent diagnosis of AIDS.

** Adjusted for sex and the other factors statistically significant in univariate analysis.

*** The youngest person who met the inclusion criteria was 19 years old.

**** ≤Primary: illiterate or primary school, Secondary: middle school, high school or technical secondary school.

MSM = men who had sex with men; IDU = injection drug user; HC = heterosexual contact with a high-risk individual or person with HIV infection or AIDS Blood = blood transfusion or blood products; AIDS = acquired immunodeficiency syndrome; HIV = human immunodeficiency virus; CD4^+^ T-cell count = CD4^+^ T-cell count within 6 months at diagnosis (cell/µL); OR = odds ratio; CI = confidence interval.

Table 6 shows the results of the univariate and multivariate Cox regression analyses of factors associated with survival. The univariate analysis demonstrated that factors, including age at diagnosis, transmission categories, marital status, educational level, CD4^+^ T-cell count within 6 months at diagnosis, concurrent HIV/AIDS diagnosis, and HAART, had significant influence on AIDS survival. The results of the univariate analysis indicated significant differences among age at diagnosis, transmission categories, marital statuses, educational level, CD4^+^ T-cell count within 6 months at diagnosis, concurrent HIV/AIDS diagnosis, and HAART. Age might be a risk factor for death (*P* for trend = 0. 01), whereas education turned out to be a protective factor (*P* for trend <0.001). The risk of death was inversely related to the number of CD4^+^ T-cells within 6 months at diagnosis (*P* for trend <0.001). The results of the multivariate analysis after adjustment for factors statistically significant in univariate analysis (plus sex) showed that AIDS patients with diagnosis ages between 50 and 59 years presented a 1.60-fold higher risk of death (*HR* = 2.60, *95% CI*, 1.18–5.72, *P* = 0.017) compared with those aged 19–29 years. With regard to CD4^+^ T-cell count within 6 months at diagnosis, AIDS patients with more number of CD4^+^ T-cells within 6 months at diagnosis had lower risk of death (*HR* = 0.29 for 50- vs <50, *95% CI*, 0.15–0.59, *P* = 0.001). The HAART reduced substantially the risk of death (*HR* = 0.02, *95% CI*, 0.01–0.04, *P = *7.25×10^−25^).

**Table 6:** HRs and 95% CIs of death among AIDS patients, Cox proportional hazard model results according to demographic factors and selected variables at AIDS diagnosis, Wuhan, China.

Characteristics	Persons with AIDS diagnosis*			
	*HR* (*95% CI*)	*P* value	Adjust *HR* (*95% CI*)**	*P* value
**Sex**		0.458		0.963
male	1.00		1.00	
female	1.18 (0.77–1.80)		0.99 (0.59–1.66)	
**Age(years)**		0.005		0.152
19–***	1.00		1.00	
30–	0.76 (0.41–1.40)	0.377	1.41(0.67–2.95)	0.362
40–	1.25 (0.69–2.27)	0.469	1.61(0.74–3.47)	0.227
50–	2.09 (1.16–3.77)	0.014	2.60 (1.18–5.72)	0.017
≥60	1.85 (0.83–4.10)	0.130	2.17 (0.80–5.89)	0.128
*χ^2^* for trend	6.55	0.010		
**Transmission category**		3.58×10^−4^		0.194
MSM	1.00		1.00	
HC	6.16 (2.47–15.34)	9.35×10^−5^	1.23 (0.44–3.43)	0.687
Blood	7.94 (2.98–21.15)	3.42×10^−5^	0.95 (0.30–2.99)	0.932
IDU	5.69 (1.94–16.67)	0.002	0.44 (0.13–1.49)	0.185
Unknown/other	14.45 (3.88–53.88)	6.97×10^−5^	1.18 (0.27–5.18)	0.825
**Marital status**		0.002		0.588
Single	1.00		1.00	
Married	2.63 (1.51–4.56)	0.001	0.67 (0.31–1.43)	0.302
Widowed/divorced	1.79 (0.96–3.32)	0.066	0.71 (0.31–1.64)	0.426
**Education** ****		2.21×10^−4^		0.275
≤Primary	1.00		1.00	
Secondary	0.65 (0.40–1.04)	0.072	1.24 (0.73–2.09)	0.427
≥College/university	0.17 (0.06–0.44)	2.28×10^−4^	0.80 (0.27–2.37)	0.685
Unknown/other	1.89 (0.77–4.65)	0.164	2.46 (0.88–6.87)	0.087
*χ^2^* for trend*****	18.93	<0.001		
**CD4^+^ T–cell count (cell/µL)**		2.14×10^−10^		5.01×10^−6^
<50	1.00		1.00	
50–	0.26 (0.14–0.49)	1.87×10^−5^	0.29 (0.15–0.59)	0.001
≥200	0.05 (0.01–0.38)	0.004	0.14 (0.02–1.12)	0.064
Unknown	1.64 (1.06–2.54)	0.026	1.51 (1.00–2.69)	0.051
*χ^2^* for trend*****	31.54	<0.001		
**AIDS at HIV infection diagnosis**		1.25×10^−5^		0.981
No	1.00		1.00	
Yes	3.11 (1.87–5.18)		0.99 (0.55–1.78)	
**HAART**		2.42×10^−30^		7.25×10^−25^
No	1.00		1.00	
Yes	0.02 (0.01–0.04)		0.02 (0.01–0.04)	

Data reported to Centers for Disease Control and Prevention from 1999 to February 29, 2012.

* HIV infection diagnosed with or without a concurrent diagnosis of AIDS.

** Relative risk adjusted for sex and the other factors statistically significant in univariate analysis.

*** The youngest person who met the inclusion criteria was 19 years old.

**** ≤Primary: illiterate or primary school, Secondary: middle school, high school or technical secondary school.

***** Unknown exclude.

MSM = men who had sex with men; IDU = injection drug user; HC = heterosexual contact with a high-risk individual or person with HIV infection or AIDS Blood = blood transfusion or blood products; AIDS = acquired immunodeficiency syndrome; HIV = human immunodeficiency virus; CD4^+^ T-cell count = CD4^+^ T-cell count within 6 months at diagnosis (cell/µL); HR = hazard ratio; CI = confidence interval.

## Discussion

Quantification of the survival after AIDS and identification of characteristics present at HIV or AIDS diagnosis offered valuable information for the better understanding about the progression of HIV infection. Our study addressed such issues and found that transmission categories and age at diagnosis were related to progression to AIDS after HIV diagnosis. Besides, Survival after AIDS diagnosis differed among age at AIDS diagnosis and number of CD4^+^ T-cells within 6 months at diagnosis (cell/µL). HAART reduced the risk of progression to AIDS after HIV diagnosis and death after AIDS diagnosis.

From the surveillance data, 74.86% of the Wuhan native AIDS patients survived 5 years after AIDS diagnosis. The one-year-survival, two-year-survival and five-year-survival were higher than that in a study conducted in Italy (83.15% vs 80.6%, 81.03% vs 75.2% and 74.86% vs 66.4%) [22]. The different economic and health care conditions between China and Italy might probably contribute to the different survival. The AIDS-related mortality decreased statistically significantly each year from 2005 onward (*P*<0.05), compared with that in 2003, which was mostly attributable to the NFATP [9]. The reduction in mortality achieved by the NFATP was consistent with results seen in other regions and countries [23], [24], [25], [26], [27], [28].

The findings of the multivariate Cox proportional hazards regression analysis revealed the potential disparities in disease progression among persons with an HIV or AIDS diagnosis. We found that excess mortality risk exist in AIDS patients with more severe disease (lower CD4^+^ T-cell count at AIDS diagnosis). Consistently with previous evidence, disease severity was proved as an independent risk factor for poor survival [4], [29], [30], whereas, HAART acted as an independent preventive factor for delaying progression to AIDS and prolonging survival for its role in improving clinical symptoms of AIDS patients, reducing HIV RNA concentration, and maintaining immune function [4], [5], [6], [31].

In the first edition (2005) of the China Free ART Manual, the CD4^+^ T-cell count criterion for treating adult patients was <200 cells/µL, and increased to <350 cells/µL in the revised (2008) version [16], [17]. As a result, more people might receive HAART. Unfortunately, 61.46% of the HIV infectors in our study received a concurrent diagnosis of AIDS which was defined by a CD4^+^ T-cell count<200 cells/µL or by an AIDS-defining illness. 32.87% AIDS patients had very low CD4^+^ T-cell counts at AIDS diagnosis (<50 cells/µL). HIV diagnosis at advanced disease may indicate rapid disease progression or late diagnosis in the disease process (testing may be motivated by symptoms). Because of social or economic factors, HIV infectors might be concurrently diagnosed with AIDS at low CD4^+^ T-cell counts for lack of adequate care to monitor the infection and institute treatment [13]. Other factors related to receipt of treatment such as health insurance, type of insurance or competing subsistence needs [32], [33], might also partly explain the differences in survival.

Older patients presented increased risk for disease progression in our analyses, which might be caused by diminished immune function and recovery and higher comorbidity [34], [35], [36], [37], [38]. An elevated risk of death associated with older age at diagnosis (*HR* = 1.49; *95% CI*, 1.34–1.66 per 10-year increase) was found in the UK population in 2008 [8], which may provide some evidence for our results. Evidence from several studies [39], [40] showed that for a given CD4^+^ T-cell count or viral load, older patients had a greater risk for progressing to AIDS than younger ones. However, adherence to treatment appeared to be higher among older compared with younger HIV infectors [41]. Moreover, elderly cases had increased significantly in recent years and thus more attention should be paid to these populations [42].

Decreased educational level was associated elsewhere with increased mortality [43], [44]. Inadequate care for knowledge about HIV/AIDS among the lower educational level groups may delay an AIDS diagnosis until symptoms appeared [45]. Reports of disparities in HIV survival related to sex have been mixed, with some studies reporting higher survival among female individuals [36], [46], some reporting it among male individuals [47], or no difference [38], [48], [49]. Consistent with a previous study [50], IDUs presented less likely to progress to AIDS following HIV diagnosis compared with MSMs, which might be related to the younger age of IDUs compared with that of MSMs. The results of univariate Cox regression analyses showed that patients transmitted by Blood suffered poorer disease progression than by other transmission category which was consistent with a previous study conducted in Mexico [51].

Previous studies investigated delayed diagnosis of HIV by using varying time frames (ranging from AIDS diagnosis within one to twelve months of HIV diagnosis) [52], [53], [54], [55], and found that men and older persons were more likely to have been diagnosed late compared with women or younger persons. The access or adherence to treatment or late diagnosis in the disease process may be responsible for the disparities we observed in progression to AIDS following HIV diagnosis. Late presentation for HIV infection diagnosis was associated with a substantially higher risk of mortality and morbidity, which argued for early diagnosis and treatment of HIV infection before patients entered advanced stages of immunodeficiency [56].

Since our study was based on retrospective data, there were inevitable some limitations. Firstly, we did not have information on severity of disease at HIV diagnosis (CD4^+^ T-cells counts) and the virus load at AIDS diagnosis for many cases (data not shown), which might affect our results [57]. Secondly, information on treatment course and clinical events, such as the type of initial ART regimen and changes in therapeutic regimens over time, was not available in our study. Previous studies had found that immunological, virological and clinical outcomes were associated with different ART regimens [58], [59], which suggested that lack of such information may contribute to some influence of our results. Thirdly, we only used data of Wuhan native cases who were over 13 years old to assess progression to AIDS and death following HIV diagnosis, which was unsure whether the results could be extrapolated nationally due to the diversity of economy and culture among districts in China.

## Conclusions

In summary, progression to AIDS and death following HIV diagnosis differed in age at diagnosis, transmission categories and CD4^+^ T-cell counts. HAART had significantly delayed the progression to AIDS following HIV diagnosis and improved the survival of AIDS patients. Thus effective interventions should target those at higher risk for morbidity or mortality, ensuring early diagnosis and timely treatment to slow down the disease progression. In future, a prospective study with larger size of sample should be carried out to obtain more information about the AIDS progression.
